# Building interest in psychiatry: Could peer-to-peer learning be a way forward in improving engagement in psychiatric education amongst medical students?

**DOI:** 10.1192/j.eurpsy.2021.1585

**Published:** 2021-08-13

**Authors:** G. Cole

**Affiliations:** Medical School, University of Bristol, Bristol, United Kingdom

**Keywords:** psychiatry, training, peer-to-peer, Medical Education

## Abstract

**Introduction:**

Stigma, stereotypes, and preconceptions have meant psychiatry has been subject to poor engagement from medical students when compared to other specialties. Whilst efforts have been made to understand reasons for this and formulate strategies to build interest, the problem still exists.

**Objectives:**

This piece explores whether giving those with a passion for psychiatry a platform to share this could gradually but positively influence their peers and thus, be a potential way to drive engagement in psychiatry as a career.

**Methods:**

Advanced literature searches explored items such as engagement in psychiatry and benefits of peer-to-peer education. CASP checklists facilitated selection and appraisal of literature for use in this discussion. Key themes were identified and used to formulate suggestions for the use of peer-to-peer teaching in building interest in psychiatry.

**Results:**

Thematic analysis of the data found 4 main themes relating to engagement in psychiatry. Current strategies to improve this have varying impact and include clinical exposure, using patients with lived experience in learning and enrichment activities, whilst the main negative influence is a long-standing stigma and stereotype around psychiatry. Three themes regarding the relevant benefits of peer-to-peer teaching were found, being peer-to-peer connection, peer influence and means to overcome stigma.

**Conclusions:**

Three key strategies for the use of peer to peer learning are suggested. These are ‘learning from students with lived experience’, ‘peer-teaching from passionate students prior to clinical exposure’ and ‘using peer learning to initially introduce topics in psychiatry in a relatable manor’.

